# Treatment of Erysipelas with Diphtheria Antitoxin

**Published:** 1911-09

**Authors:** 


### Treatment of Erysipelas with Diphtheria Antitoxin

POLAK (*Klinisch-therapeutische Wochenschrift,* Jahrg. 18, No. 17) has used diphtheria serum in the treatment of erysipelas for the past two years. In most cases the results have been surprisingly good. The fever sinks to normal in twenty-four to thirty-six hours and does not rise again, nor do any complications occur. Coincidentally with the fall of temperature the edema and redness disappear, and the appetite returns. In cases in which the temperature did not fall in two days a second injecion brought it down promptly. Injection was made in all cases, even when treatment promised but little or when there was doubt as to the diagnosis between erysipelas and lymphangitis or other affection.

The experience of the author has been that of all means of treating erysipelas, that by means of diphtheria serum gives by far the most certain results. For the purpose of comparison, serum was injected in puerperal infection, mastitis, osteomyelitis, phlegmon, and furunculosis, conditions supposed to be due to streptococcus infection, but without any appreciable good results.—*Therapeutic Gazette.*

